# Gene expression changes in vastus lateralis muscle after different strength training regimes during rehabilitation following anterior cruciate ligament reconstruction

**DOI:** 10.1371/journal.pone.0258635

**Published:** 2021-10-14

**Authors:** Birgit Friedmann-Bette, Holger Lornsen, Mario Parstorfer, Thomas Gwechenberger, Francesca Profit, Marc-André Weber, Alexander Barié

**Affiliations:** 1 Department of Sports Medicine, Medical Clinic, University Hospital Heidelberg, Heidelberg, Germany; 2 Olympic Training Center, Heidelberg, Germany; 3 Department of Orthopaedic and Trauma Surgery, Center for Orthopaedics, Trauma Surgery and Spinal Cord Injury, University Hospital Heidelberg, Heidelberg, Germany; 4 Department of Diagnostic and Interventional Radiology, University Hospital Heidelberg, Heidelberg, Germany; 5 Institute of Diagnostic and Interventional Radiology, Pediatric Radiology and Neuroradiology, University Medical Center Rostock, Rostock, Germany; 6 Center for Joint Surgery and Sport Injuries, Sportopaedie, Clinic St. Elisabeth, Heidelberg, Germany; University of Tennessee Health Science Center College of Graduate Health Sciences, UNITED STATES

## Abstract

Impaired muscle regeneration has repeatedly been described after anterior cruciate ligament reconstruction (ACL-R). The results of recent studies provided some evidence for negative alterations in knee extensor muscles after ACL-R causing persisting strength deficits in spite of the regain of muscle mass. Accordingly, we observed that 12 weeks of concentric/eccentric quadriceps strength training with eccentric overload (CON/ECC^+^) induced a significantly greater hypertrophy of the atrophied quadriceps muscle after ACL-R than conventional concentric/eccentric quadriceps strength training (CON/ECC). However, strength deficits persisted and there was an unexpected increase in the proportion of slow type I fibers instead of the expected shift towards a faster muscle phenotype after CON/ECC^+^. In order to shed further light on muscle recovery after ACL-R, the steady-state levels of 84 marker mRNAs were analyzed in biopsies obtained from the vastus lateralis muscle of 31 subjects before and after 12 weeks of CON/ECC^+^ (n = 18) or CON/ECC strength training (n = 13) during rehabilitation after ACL-R using a custom RT^2^ Profiler PCR array. Significant (p < 0.05) changes were detected in the expression of 26 mRNAs, several of them involved in muscle wasting/atrophy. A different pattern with regard to the strength training mode was observed for 16 mRNAs, indicating an enhanced hypertrophic stimulus, mechanical sensing or fast contractility after CON/ECC^+^. The effects of the type of autograft (quadriceps, QUAD, n = 19, or semitendinosus tendon, SEMI, n = 12) were reflected in the lower expression of 6 mRNAs involved in skeletal muscle hypertrophy or contractility in QUAD. In conclusion, the greater hypertrophic stimulus and mechanical stress induced by CON/ECC^+^ and a beginning shift towards a faster muscle phenotype after CON/ECC^+^ might be indicated by significant gene expression changes as well as still ongoing muscle wasting processes and a negative impact of QUAD autograft.

## Introduction

Muscle regeneration after anterior cruciate ligament reconstruction (ACL-R) is impaired. Even with guided rehabilitation, atrophy and strength deficits of the quadriceps femoris muscle were reported up to one year after ACL surgery [1–3] and even longer in subjects with chronically insufficient ACL [4]. Prolonged quadriceps weakness could only partially be explained by rather small reductions in quadriceps muscle mass indicating a discrepancy between the regain in quadriceps muscle mass and quadriceps strength [2,5,6]. This discrepancy might partly be due to impaired neuromuscular function, which is observed after ACL injury [7,8]. However, it might also be explained by altered muscle quality or phenotype, respectively. Increases in collagen content, in the abundance of fibroblasts as well as in fibrogenic/adipogenic progenitor cells and a reduction in satellite cell number were found in biopsies obtained from the vastus lateralis muscle after ACL-injury [9–11]. Furthermore, incomplete recovery of fiber type distribution, fiber cross sectional area, capillarization and mitochondrial respiration was reported after standard rehabilitation training following ACL-R [4]. Recently, we observed a significant increase in quadriceps muscle mass after 12 weeks of supervised progressive strength training performed subsequently to the early rehabilitation period after ACL-R in recreational athletes. The increase in quadriceps muscle mass was significantly enhanced, when the strength training was conducted as concentric/eccentric strength training with eccentric overload (CON/ECC^+^) compared with conventional concentric/eccentric strength training (CON/ECC). Besides the discrepancy between quadriceps muscle mass and strength, a significant increase in the proportion of type 1 fibers after CON/ECC^+^ was a surprising result [5]. So far, CON/ECC^+^ had been found to be especially beneficial in enhancing strength, power and speed performance, which are the prevailing characteristics of a fast muscle phenotype [12–14]. With regard to the indications for reduced muscle quality observed in other studies on muscle regeneration after ACL injury, we wondered if the development of a slower muscle phenotype after CON/ECC^+^ might be caused by altered quality of the atrophied quadriceps muscle.

In an attempt to 1) investigate the adaptation to CON/ECC^+^ in comparison to CON/ECC strength training and to 2) shed further light on the impaired muscle recovery after ACL-R, we measured the steady state expression of selected marker mRNAs in the vastus lateralis biopsies obtained from recreational athletes in our previous investigation [5]. The biopsies were performed before and after 12 weeks of either conventional leg-press training or leg-press training with eccentric overload on a computer-guided device taking place subsequently to the early rehabilitation period following ACL-R. Using a custom RT^2^ Profiler PCR array, training-induced changes in the steady state expression of 84 key genes involved in skeletal muscle myogenesis and hypertrophy, skeletal muscle wasting/atrophy, skeletal muscle contractility, and skeletal muscle autocrine signaling and/or energy sensing were analyzed. We hypothesized that 1) differences in the adaptation to the two different training regimes would emerge in the steady state expression of genes involved in skeletal muscle myogenesis and hypertrophy as well as in skeletal muscle contractility and that 2) the 12-wk strength training would also affect the expression of some genes involved in muscle wasting and muscle atrophy in the still dysfunctional muscle after ACL-R.

## Materials and methods

### General design

This study is part of an investigation on muscle recovery after ACL-R. Some data have recently been published and detailed descriptions of the strength training programs, strength testing, the MRI-procedures and the determination of fiber type distribution and fiber cross-sectional areas with immunohistochemistry are provided elsewhere [5]. Briefly, recreational athletes, who underwent standardized ACL-R with either quadriceps tendon (QUAD) or semitendinosus tendon autograft (SEMI) were asked to participate in the study at the time point of ACL surgery. They had to be otherwise healthy and met the inclusion criteria (age, 18–35 yr, regular recreational activity with at least three work-outs per week in a variety of sports, first traumatic ACL rupture within the last year without further serious knee injury or signs of arthrosis). After finishing the regularly scheduled 12-wk early rehabilitation with physiotherapy treatment, the subjects were randomly assigned to 12-wk supervised progressive quadriceps strength training performed twice a week (Monday and Thursday or Tuesday and Friday, respectively), either as conventional one-legged concentric/eccentric (CON/ECC) leg-press training (Compass; Proxomed, Wolfratshausen, Germany) or as one-legged computer-guided leg-press training (IsoMed 2000; D&R Ferstl, Hemnau, Germany) with eccentric overload. All subjects performed 6 sets of 8 repetitions at the 8 repetition maximum, i.e. the load was chosen to cause exhaustion after 8 repetitions on both training devices. The series of 8 repetitions were separated by 90 s rest. In the case of CON/ECC, load was controlled by weight. The same absolute load was applied in the concentric as well as the eccentric phases. After 6, 12, and 18 training sessions, the applied weight was adjusted for eight-repetition maximum. That led to an average of 2.2-fold increase in the load throughout the study. In CON/ECC^+^, load was controlled by velocity. At the beginning of the training period, the leg-press plate moved with the same velocity (200 mm ∙ s^-1^) during the concentric and eccentric phases. After 6, 12, and 18 training sessions, velocity was successively increased during the eccentric phase and decreased during the concentric phase to 400 and 100 mm ∙ s^-1^, respectively. Subjects pressed against the leg-press plate moving away from the body during the concentric phase. During the eccentric phase, the leg-press plate moved toward the subjetcs´ body against the resisting extensor muscle group. Subjects were instructed to develop maximal force onto the leg-press plate during the 8 repetitions in the concentric as well as the eccentric phase. At the beginning of the training period, the workload during the concentric contraction was 1399 ± 439 J and increased to 1850 ± 517 J at the end of the 12-wk strength training. The workload during the eccentric contraction increased from 1595 ± 504 J to 2299 ± 698 J. On the average, the eccentric load was 1.14-fold higher than the concentric load at the beginning and 1.24-fold higher at the end of the 12-wk training period. Throughout the study, the subjects were not allowed to perform any training except the two sessions of supervised strength training. Furthermore, they had to refrain from the consumption of any nutritional supplements, especially from protein or creatine supplements. Before and after the 12-wk strength training, muscle biopsies were obtained from the vastus lateralis muscle of the injured leg, 5–8 days after the last exercise bout.

### Participants

In 31 out of the 37 recreational athletes (28 males, 3 females) from whom muscle biopsies had been obtained in the already published study, there was enough biopsy material left over after the immunohistochemical analyses for RT-PCR analyses (CON/ECC: n = 13, age, 27 ± 5 years; height, 179 ± 6 cm; weight, 80.1 ± 9.3 kg; 4 SEMI, 9 QUAD; CON/ECC^+^: n = 18; age, 24 ± 4 years; height, 182 ± 7 kg; weight, 86.5 ± 16.4 kg; 8 SEMI, 10 QUAD). The changes in muscle cross sectional area (MCSA), fiber cross sectional area (FCSA) and fiber type distribution after the 12-wk strength training in the smaller data subset of the present study were similar to what was previously described [5]. Briefly, there was a significantly greater increase in muscle cross sectional area (MCSA) in the CON/ECC^+^ compared with the CON/ECC training group. Fiber cross sectional area of all fiber types was significantly increased after the 12-wk strength training without significant differences between the training groups. Surprisingly, only after the 12-wk CON/ECC^+^ training, the proportion of type I fibers was significantly increased S1 Fig. The study was approved by the ethics committee of the local Medical Faculty and all participants provided written, informed consent prior to participation. All procedures of the present study were conducted in accordance with the Declaration of Helsinki.

### Skeletal muscle biopsies

Biopsies were obtained from the same region at mid-thigh level of the injured leg under local anesthesia, using the Bergström technique [15]. The muscle tissue was immediately freed from blood and visible connective tissue, rapidly frozen in isopentane cooled by liquid nitrogen and subsequently stored at -80°C. To avoid residual effects from preceding biopsies, biopsy sites were spaced apart by at least 1 cm in the longitudinal direction.

#### RNA extraction and reverse transcription

For RNA extraction, 25 μm sections were cut on a cryostat. The area of the cutting surface was estimated by planimetry. The number of sections cut was adjusted to reach approximately 10 mm^3^ (equivalent to 10 mg tissue, assuming an approximate density of 1). From these sections, total RNA was isolated using a modification of the Qiagen micro-protocol for skeletal muscle as described previously (Qiagen, Hilden, Germany) [13,14,16,17]. RNA concentration and purity were determined by A260 and A280 measurements (Colibri Microvolume Spectrometer, Titertek Berthold, Pforzheim, Germany). cDNA was synthesized from 100 ng of total RNA in a 20 μl reaction using Qiagen RT^2^ First Strand Kit according to the manufacturer´s protocols (Qiagen, Hilden, Germany).

#### Real-time PCR

The cDNA was mixed with RT^2^ SYBR Green Mastermix (Qiagen, Hilden, Germany) according to the manufacturer´s protocols. The mixture was loaded into the 96-well RT^2^ Profiler^TM^ PCR Array *Human Skeletal Muscle*: *Myogenesis and Myopathy* (Qiagen, Hilden, Germany, cat. no.: 330231 PAHS-099ZA) to examine the mRNA levels of 84 genes (complete list shown in Table 1) using the LightCycler^TM^ 480 (Roche Applied Systems, Mannheim, Germany). Analysis of PCR array data was done according to manufacturer´s instructions. Each array contained 5 housekeeping genes [actin, beta (ACTB, NM_001101); beta-1-microglobulin (B2M, NM_004048); glyceraldehyde-3-phosphate dehydrogenase (GAPDH, NM_002046); hypoxanthine phosphoribosyltransferase 1 (HPRT1, NM_000194); Ribosomal protein, large P0 (RPLP0, NM_001002)]. Because of high variation between the samples, HPRT1 was unsuitable as housekeeping gene. Therefore, sample data were normalized against ACTB, B2M, GAPDH and RPLP0 only. Several negative controls were included in each run. The transcript level of each candidate gene was quantified according to the ΔΔCt method. Ct values >35 were not included in the analysis and considered as negative.

**Table 1:** RT^2^ Profiler^TM^ PCR array human skeletal muscle: Myogenesis & Myopathy.

Target mRNA	Symbol	Gene-bank accession no.	Involvement
Actin, alpha 1	ACTA1	NM_001100	Titin complex, contractility; myogenesis/hypertrophy
Actinin, alpha 3	ACTN3	NM_001104	Titin complex, contractility
Activin A receptor, type IIb	ACVR2B	NM_001106	Hypertrophy
Adiponectin	ADIPOQ	NM_004797	Autocrine signaling; metabolic syndrome
Adrenergic, beta-2-, receptor, surface	ADRB2	NM_000024	Myogenesis/hypertrophy
Agrin	AGRN	NM_198576	Myogenesis
V-akt murine thymoma viral oncogene homolog 1	AKT1	NM_005163	Dystrophy
V-akt murine thymoma viral oncogene homolog 1	AKT2	NM_001626	Dystrophy
ATPase, Ca^++^ transport-ting, cardiac muscle fast twitch 1	ATP2A1	NM_173201	Contractility
B-cell CLL/lymphoma 2	BCL2	NM_000633	Myogenesis
Bone morphogenetic protein4	BMP4	NM_130851	Myogenesis
Calcium/calmodulin-dependent protein kinase II gamma	CAMK2G	NM_001222	Dystrophin-glycoprotein complex, contractility
Calpain 2, (m/II), large subunit	CAPN2	NM_001748	Myogenesis; atrophy
Calpain 3 (p94)	CAPN3	NM_0173090	Dystrophin-glycoprotein complex, contractility
Caspase 3, apoptosis-related cysteine peptidase	CASP3	NM_004346	Atrophy
Calpastatin	CAST	NM_001042440	Myogenesis
Caveolin 1, caveolae protein, 22kDa	CAV1	NM_001753	Myogenesis
Caveolin 3	CAV3	NM_001234	Dystrophin-glycoprotein complex, contractility
Crystallin, alpha B	CRYAB	NM_001885	Titin complex, contractility
Citrate synthase	CS	NM_004077	Energy metabolism, contractility
Catenin, beta 1, 88kDa	CTNNB1	NM_001904	Myogenesis
Dystroglycan 1	DAG1	NM_004393	Dystrophin-glycoprotein complex, contractility
Desmin	DES	NM_001927	Titin complex, contractility
Dystrophin	DMD	NM_000109	Myogenesis; dystrophin-glycoprotein complex, contractility
Dystrophia myotonica-protein kinase	DMPK	NM_004409	Contractility
Dysferlin	DYSF	NM_003494	Dystrophin-glycoprotein complex, contractility
F-box protein 32	FBXO32	NM_058229	Atrophy; dystrophy
Forkhead box O1	FOXO1	NM_002015	Atrophy
Forkhead box O3	FOXO3	NM_001455	Atrophy
Histon deacetylase 5	HDAC5	NM_005474	Myogenesis
Hexokinase 2	HK2	NM_000189	Energy metabolism, contractility
Insulin-like growth factor 1	IGF1	NM_000618	Myogenesis; hypertrophy; autocrine signaling
Insulin-like growth factor 2	IGF2	NM_000612	Autocrine signaling
Insulin-like growth factor binding protein 3	IGFBP3	NM_000598	Myogenesis
Insulin-like growth factor binding protein 5	IGFBP5	NM_000599	Myogenesis; hypertrophy
Inhibitor of kappa light polypeptide gene enhancer in B-cells, kinase beta	IKBKB	NM_001556	Contractility
Interleukin 1, beta	IL1B	NM_000576	Dystrophy
Interleukin 6	IL6	NM_000600	Autocrine signaling
Leptin	LEP	NM_000230	Autocrine signaling; metabolic syndrome
Lamin A/C	LMNA	NM_005572	Dystrophin-glycoprotein complex, titin complex, contractility
Mitogen-activated protein kinase 1	MAPK1	NM_002745	Dystrophin-glycoprotein complex, titin complex, contractility; dystrophy
Mitogen-activated protein kinase 14	MAPK14	NM_001315	Dystrophy
Mitogen-activated protein kinase 3	MAPK3	NM_002746	Dystrophy
Mitogen-activated protein kinase 8	MAPK8	NM_002750	Dystrophy
Myoglobin	MB	NM_005368	Contractility
Myocyte enhancer factor 2C	MEF2C	NM_002397	Myogenesis
Matrix metallopeptidase 9	MMP9	NM_004994	Dystrophy
Myostatin	MSTN	NM_005259	Myogenesis; hypertrophy; titin complex, contractility; autocrine signaling
Muscle, skeletal, receptor tyrosine kinase	MUSK	NM_005592	Myogenesis
Myogenic factor 5	MYF5	NM_005593	Myogenesis
Myogenic factor 6	MYF6	NM_002469	Myogenesis; hypertrophy
Myosin heavy chain 1	MYH1	NM_005963	Contractility
Myosin heavy chain 2	MYH2	NM_017534	Contractility
Myogenic differentiation 1	MYOD1	NM_002478	Myogenesis; hypertrophy
Myogenin	MYOG	NM_002479	Myogenesis
Myotilin	MYOT	NM_006790	Titin complex, contractility
Nebulin	NEB	NM_004543	Titin complex, contractility
Nuclear factor of kappa light polypeptide gene enhancer in B-cells 1	NFKB1	NM_003998	Dystrophy
Nitric oxide synthase 2	NOS2	NM_000625	Atrophy
Paired box 3	PAX3	NM_181461	Myogenesis
Paired box 7	PAX7	NM_002584	Myogenesis
Pyruvate dehydrogenase kinase, isoenzyme 4	PDK4	NM_002612	Energy metabolism, contractility
Peroxisome proliferator-activated receptor gamma	PPARG	NM_015869	Metabolic syndrome
Peroxisome proliferator-activated receptor gamma, coactivator 1 alpha	PPARGC1A	NM_013261	Atrophy; metabolic syndrome
Peroxisome proliferator-activated receptor gamma, coactivator 1 beta	PPARGC1B	NM_133263	Atrophy; metabolic syndrome
Protein phosphatase 3, catalytic subunit, alpha isoenzyme	PPPCA3	NM_000944	Myogenesis
Protein kinase, AMP-activated, alpha 1 catalytic subunit	PRKAA1	NM_006251	Metabolic syndrome
Protein kinase, AMP-activated, beta 2 non-catalytic subunit	PRKAB2	NM_005399	Metabolic syndrome
Protein kinase, AMP-activated, gamma 1 non-catalytic subunit	PRKAG1	NM_002733	Metabolic syndrome
Protein kinase, AMP-activated, gamma 3 non-catalytic subunit	PRKAG3	NM_017431	Metabolic syndrome
Ras homolog gene family, member A	RHOA	NM_001644	Myogenesis
Ribosomal protein 6 kinase, 70kDa, polypeptide 1	RPS6KB1	NM_003161	Myogenesis; hypertrophy; atrophy
Sarcoglycan, alpha	SGCA	NM_000023	Dystrophin-glycoprotein complex; titin complex, contractility
Solute carrier family 2, member 4	SLCA4	NM_001042	Energy metabolism, contractility; metabolic syndrome
Transforming growth factor, beta 1	TGFB1	NM_000660	Autocrine signaling
Tumor necrosis factor	TNF	NM_000594	Dystrophy
Troponin C type 1	TNNC1	NM_003280	Contractility
Troponin I type 2	TNNI2	NM_003282	Titin complex, contractility
Troponin T type 1	TNNT1	NM_003283	Titin complex, contractility
Troponin T type 3	TNNT3	NM_006757	Titin complex, contractility
Tripartite motif containing 63	TRIM63	NM_032588	Atrophy; dystrophy; titin complex, contractility
Titin	TTN	NM_003319	Contractility
Utrophin	UTRN	NM_007124	Myogenesis; dystrophy

### Statistical analyses

Statistical tests were processed using SPSS 24.0 software for Windows (SPSS Inc., Chicago, IL) and Graph Pad Prism 8 (GraphPad Software Inc., San Diego, CA) software for Windows. The effects of strength training on the expression of the marker mRNAs were assessed using mixed analysis of covariance (ANCOVA) repeated-measures analysis with time (before and after the 12-wk training period) as within variable and training group (CON/ECC and CON/ECC^+^) as between subject variable. Type of ACL-autograft (QUAD and SEMI) was entered as covariate. First, the normal distribution of all data was checked using the Kolmogorov-Smirnov test. As this test failed for most of the marker mRNAs, log-transformed data were used for further analysis. Additionally, effect sizes were determined by partial eta-squared (ɳ_p_^2^). Post-hoc analyses were conducted where appropriate, and a Bonferroni correction was used for multiple comparisons. For all analyses, a value of p ≤ 0.05, set a priori, was considered to represent statistical significance. All data are presented as mean ± SE. R, a free software environment for statistical computing and graphics (R Core Team, 2020) was used for principle component analysis (dplyr, ggplot2) to find out to what extent the pattern of expression of genes was different between groups.

## Results

### Significantly regulated genes

Overall, 26 out of the 84 key genes, involved in skeletal muscle differentiation, function and disease-related processes, were significantly regulated after the 12-wk training period, i.e. the steady state levels of these marker transcripts were significantly changed after CON/ECC and/or CON/ECC^+^ training. However, principle component analysis did not reveal a difference in the pattern of gene expression changes between the training groups or with regard to the type of autograft.

#### Genes involved in skeletal myogenesis and skeletal muscle hypertrophy

**Fig 1:**
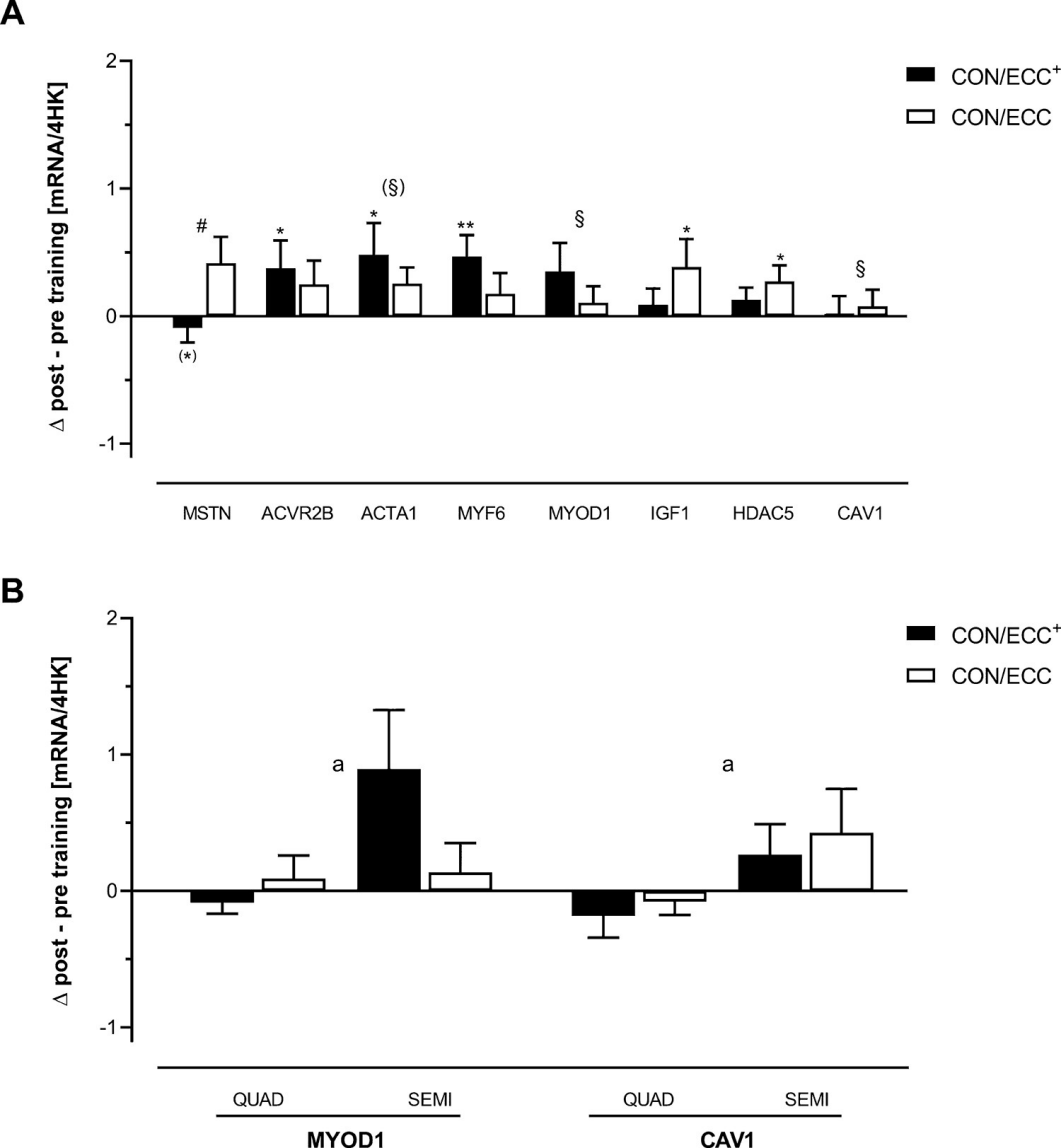
A. Significantly changed marker mRNAs encoded by genes involved in skeletal myogenesis and skeletal muscle hypertrophy. B. Significantly changed marker mRNAs encoded by genes involved in skeletal myogenesis and skeletal muscle hypertrophy significantly affected by the type of autograft. These are relative values based on an aggregate of 4 housekeeping (HK) transcripts. Mean ± SE are shown. #: Significant group x time effect (p < 0.05); §: Significant time effect (p < 0.05); (§): Tendency (p = 0.059) towards a time effect; *: p < 0.05, **: p < 0.01 compared to the respective baseline value; (*): Tendency (p = 0.072) for a difference compared to the respective baseline value; a: Significant effect of the type of autograft (p < 0.05).

For MSTN, also involved in muscle wasting and skeletal muscle contractility, a significant (p = 0.024) group x time interaction was observed in the mRNA expression (F(1,28) = 5.681, η_p_^2^ = 0.169). There was a tendency (p = 0.072) towards a decrease in MSTN-mRNA expression in the CON/ECC^+^ training group Fig 1A.

Significant main time effects were found for the mRNA expression of CAV1 (F(1,28) = 6.570, η_p_^2^ = 0.190, p = 0.016) and of MYOD1 (F(1,28) = 5.528, η_p_^2^ = 0.165, p = 0.026) Fig 1A. Type of autograft had a significant (p < 0.01) effect on the mRNA levels of both these genes with a higher expression of CAV1-mRNA after 12 weeks of CON/ECC and CON/ECC^+^ training in the SEMI than in the QUAD subjects and with a higher mRNA expression of MYOD1 in the SEMI than in the QUAD subjects Fig 1B.

Only after CON/ECC^+^ training, a significantly increased mRNA expression of MYF6 (p = 0.008), of ACTA1 (p = 0.029, also involved in skeletal muscle contractility) and of ACVR2B (p = 0.031) was found. There was, however, no significant time x group interaction for the regulation of these mRNAs. A trend towards a significant time effect was seen for ACTA1-mRNA (p = 0.059) Fig 1A.

Only after CON/ECC training, a significantly increased mRNA expression of HDAC5 (p = 0.030) and of IGF1 (p = 0.035, also involved in skeletal muscle autocrine signaling) was observed Fig 1A.

#### Genes involved in muscle wasting/atrophy

**Fig 2:**
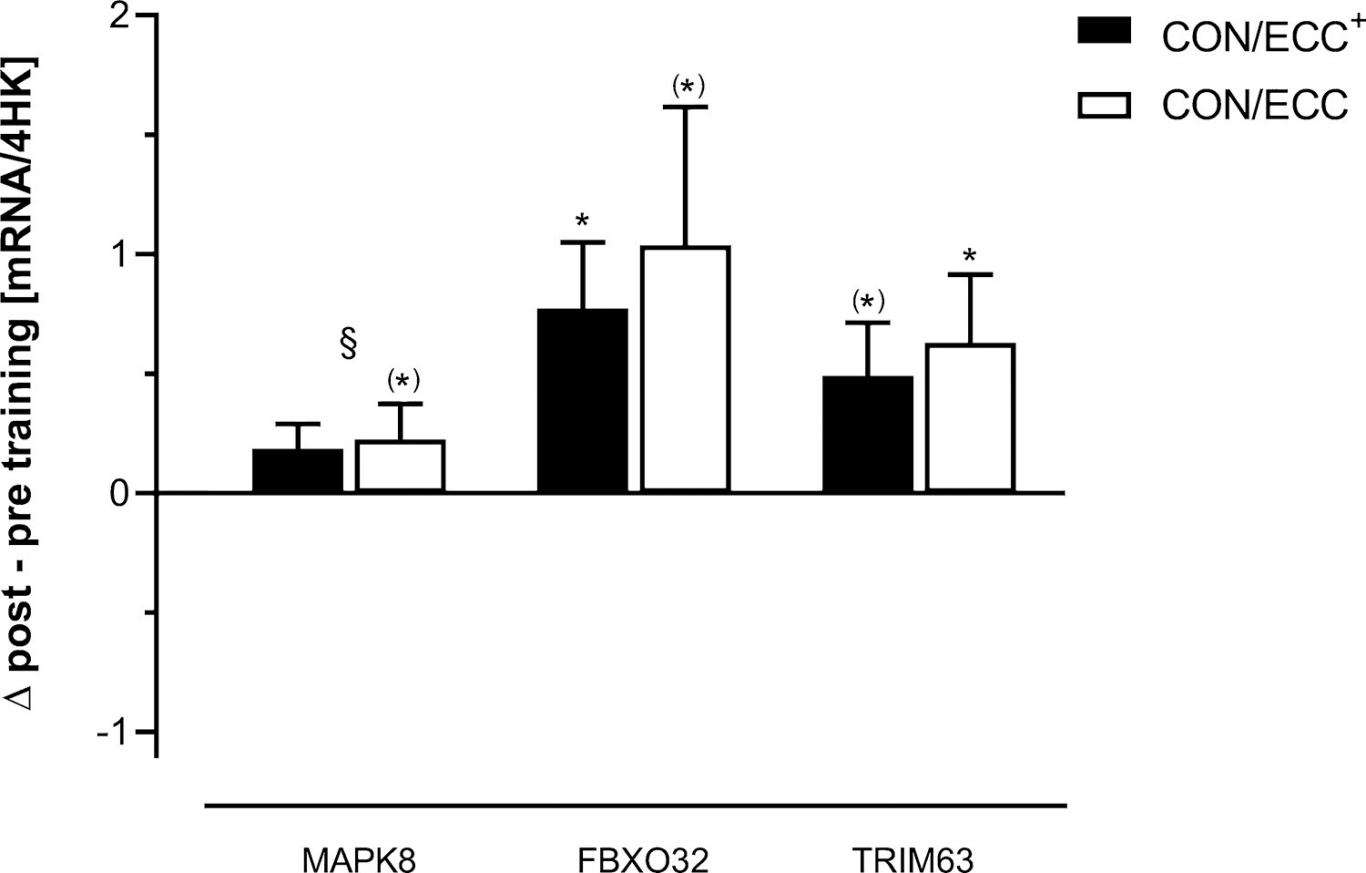
Significantly changed marker mRNAs encoded by genes involved in muscle wasting/atrophy. These are relative values based on an aggregate of 4 housekeeping (HK) transcripts. Mean ± SE are shown. §: Significant time effect (p < 0.05); *: p < 0.05; (*): Tendency (MAPK8: p = 0.068; FBXO32: p = 0.056; TRIM63: p = 0.051) for a difference compared to the respective baseline value.

A significant (p = 0.029) main time effect was observed for MAPK8-mRNA expression (F(1,28) = 5.293, η_p_^2^ = 0.159). Post hoc testing showed a tendency (p = 0.068) for an increased expression of MAPK8-mRNA after CON/ECC training only Fig 2.

mRNA expression of FBXO32 was significantly (p = 0.035) increased after CON/ECC^+^ training and tended to be increased (p = 0.056) after CON/ECC training, however, with a wide inter-individual variation Fig 2.

Expression of TRIM63-mRNA was significantly (p = 0.028) increased after CON/ECC training and tended to be increased (p = 0.051) after CON/ECC^+^ training Fig 2.

#### Genes involved in skeletal muscle contractility

**Fig 3:**
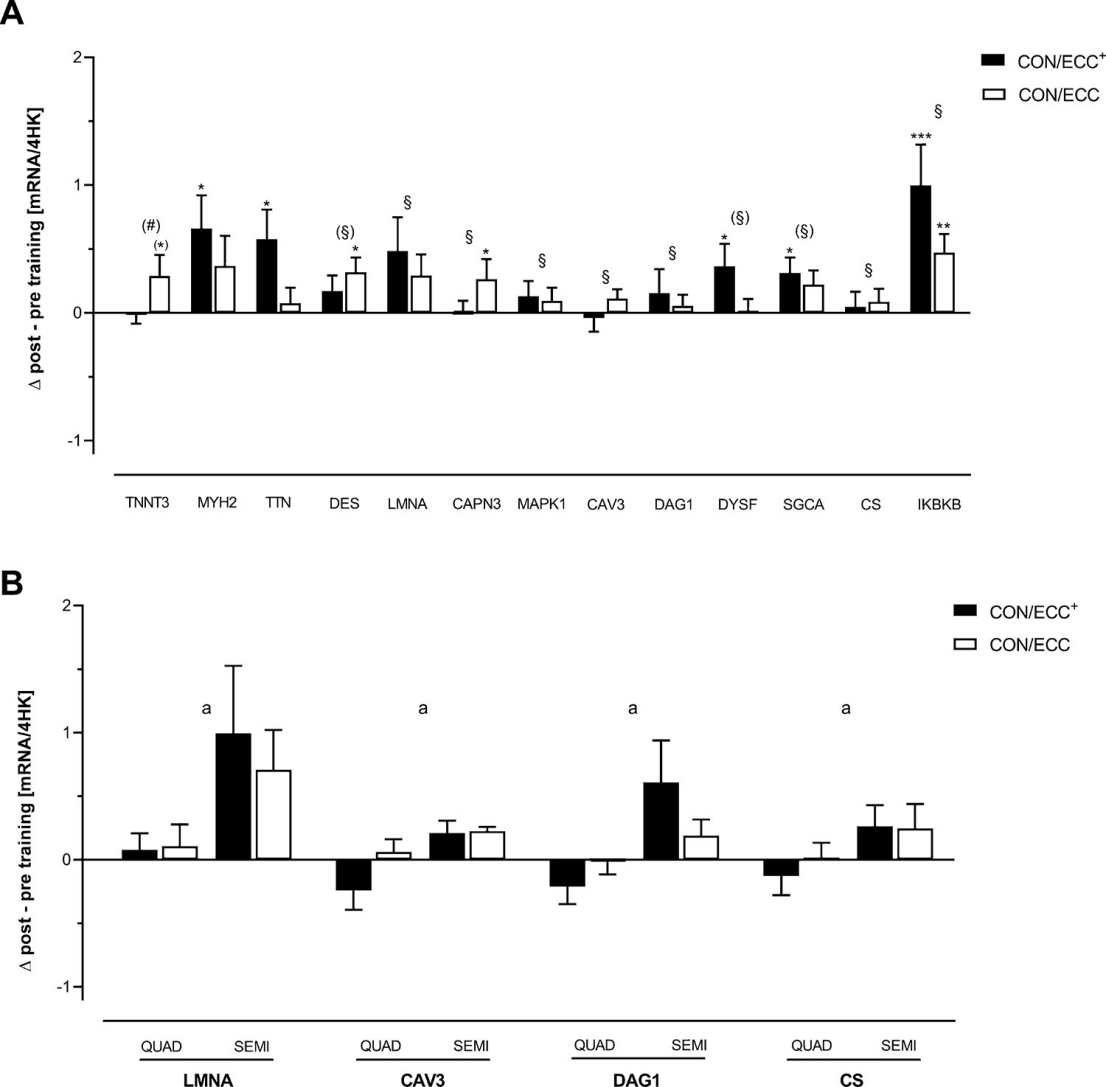
A. Significantly changed marker mRNAs encoded by genes involved in skeletal muscle contractility. B. Significantly changed marker mRNAs encoded by genes involved in skeletal muscle contractility significantly affected by the type of autograft. These are relative values based on an aggregate of 4 housekeeping (HK) transcripts. Mean ± SE are shown. (#): Tendency (p = 0.053) towards a group x time effect; §: Significant time effect (p < 0.05); (§): Tendency (DES: p = 0.066; DYSF: p = 0.062; SGCA: p = 0.067) towards a time effect; *: p < 0.05, **: p < 0.01 compared to the respective baseline value; (*): Tendency (p = 0.052) for a difference compared to the respective baseline value; a: Significant (p < 0.05) effect of the type of autograft.

There was a strong tendency (p = 0.053) for a group x time effect for TNNT3-mRNA expression (F(1,28 = 4.09, η_p_^2^ = 0.128). After CON/ECC training, there was a tendency (p = 0.052) for an increase in the expression of TNNT3 Fig 3A.

Significant main time effects were found for the mRNA expression of LMNA (F(1,28) = 6.418, η_p_^2^ = 0.186, p = 0.017), CAPN3 (F(1,28) = 5.761, η_p_^2^ = 0.171, p = 0.037) and of MAPK1 (F(1,28) = 4.802, η_p_^2^ = 0.146, p = 0.037), all belonging to the Titin Complex and the Dystrophin-Glycoprotein Complex. Post-hoc testing only revealed a significant (p = 0.029) increase in CAPN3-mRNA expression after CON/ECC training Fig 3A. Type of autograft had a significant effect on the mRNA expression of LMNA (p = 0.040, higher expression in the SEMI group irrespective of the training mode) Fig 3B.

Further main time effects were observed for the mRNA expression of CAV3 (F(1,28) = 6.970, η_p_^2^ = 0.199, p = 0.013) and of DAG1 (F(1.28) = 6.043, η_p_^2^ = 0.178, p = 0.020), both belonging to the Dystrophin-Glycoprotein Complex Fig 3A. Type of autograft had a significant effect on the expression changes of both genes (CAV3: p = 0.015, DAG: p = 0.012, respectively) with increased expression in the SEMI group in contrast to a decreased expression in the QUAD group after CON/ECC^+^ training Fig 3B.

Furthermore, a main time effect occurred for the expression of CS-mRNA (F(1,28) = 4.942, η_p_^2^ = 0.150, p = 0.034), a marker gene for energy metabolism, and of IFBKB-mRNA (F(1,28) = 6.641, η_p_^2^ = 0.192, p = 0.016). There were significant increases after CON/ECC^+^ (p < 0.001) and after CON/ECC training (p = 0.009) for IFBKB-mRNA expression Fig 3A. Type of autograft had a significant (p = 0.048) effect on the mRNA expression of CS with an increase in the SEMI group after the 12-wk training period in contrast to a decreased expression after CON/ECC^+^ training in the QUAD group Fig 3B.

Only after CON/ECC^+^ training, mRNA expression of MYH2 (p = 0.048) and of TTN (p = 0.045) was significantly increased, both involved in the Titin complex Fig 3A.

The mRNA expression of two genes belonging to the Dystrophin-Glycoprotein complex, DYSF (p = 0.022) and SGCA (p = 0.021) was also significantly increased after CON/ECC^+^ training. For both these mRNAs, there was a tendency for a main time effect (DYSF: p = 0.062, SGCA: p = 0.067) Fig 3A.

A significant (p = 0.019) increase in the mRNA expression of DES (belonging to the Titin Complex) was observed after CON/ECC training. There also was a tendency for a main time effect (p = 0.066) Fig 3A.

#### Genes involved in skeletal muscle autocrine signaling and/or energy-sensing

**Fig 4:**
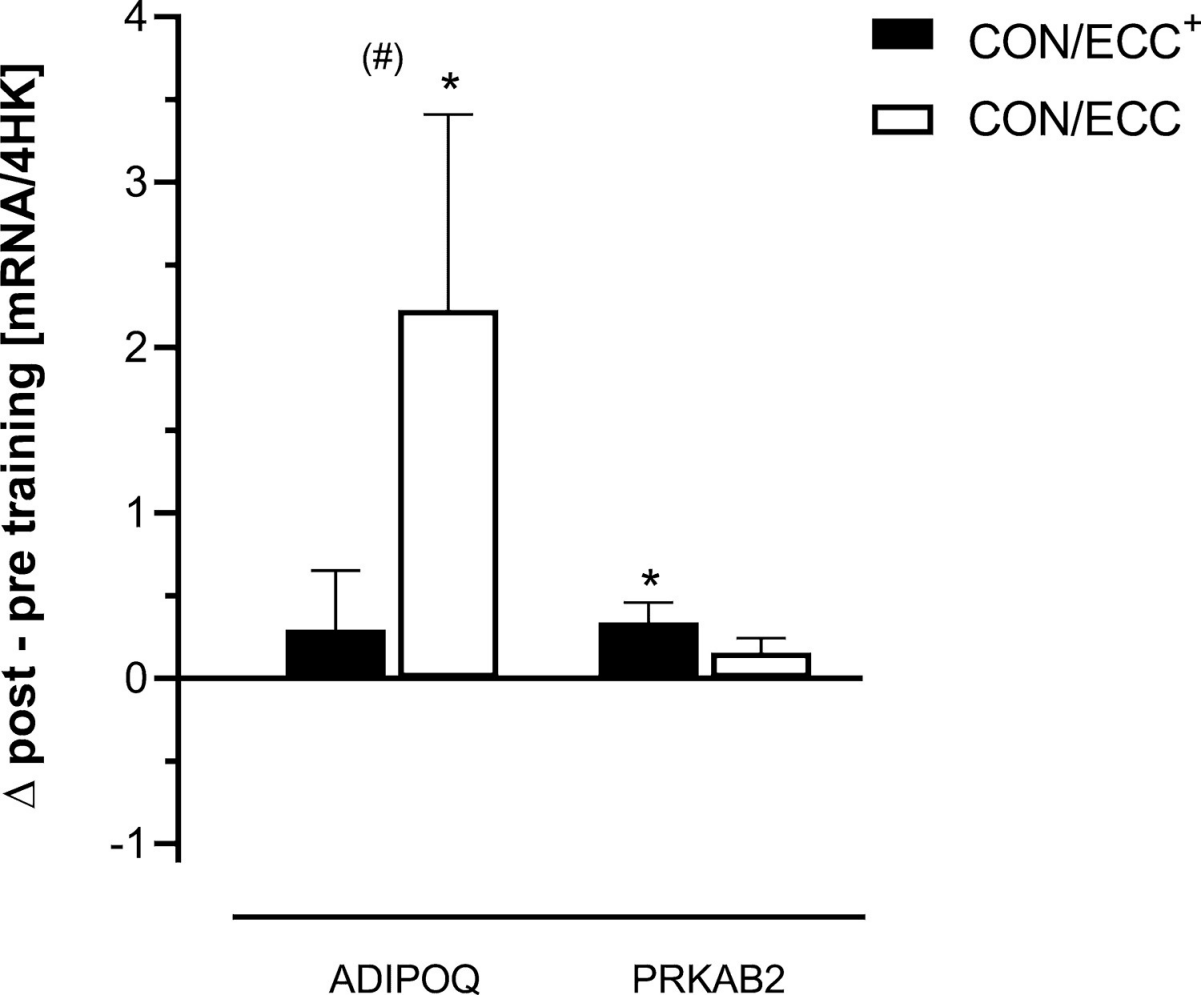
Significantly changed marker mRNAs encoded by genes involved in skeletal muscle autocrine signaling and/or energy-sensing. These are relative values based on an aggregate of 4 housekeeping (HK) transcripts. Mean ± SE are shown. (#): Tendency (p = 0.052) towards a group x time effect; *: p < 0.05, **: p < 0.01 compared to the respective baseline value.

A strong tendency (p = 0.052) for a group x time effect was observed for the expression of ADIPOQ-mRNA F(1,15) = 4.467, η_p_^2^ = 0.229). Post hoc testing revealed a significant (p = 0.011) increase in ADIPOQ-mRNA expression after CON/ECC training only Fig 4. However, gene expression of ADIPOQ could only be detected in 6 out of 13 subjects of the CON/ECC and in 12 out 18 subjects of the CON/ECC^+^ training group.

PRKAB2-mRNA expression was significantly (p = 0.002) increased after CON/ECC^+^ training only.

## Discussion

This study is part of the, to our best knowledge, first investigation on quadriceps muscle adaptation to supervised strength training during rehabilitation after ACL-R. Differences between the two different modes of the 12-wk strength training, CON/ECC^+^ and CON/ECC, emerged in a significantly different regulation of the MSTN-mRNA expression with a trend towards a decrease after CON/ECC^+^ and in some slight differences in the expression changes of further 15 mRNAs involved in skeletal myogenesis/hypertrophy and contractility. The significantly increased expression of 6 marker mRNAs involved in muscle wasting after both types of strength training added further evidence to the observation of a declined muscle quality after ACL-injury. Furthermore, effects of different types of autografts were reflected in the expression changes of 6 mRNAs involved in skeletal myogenesis/hypertrophy and contractility.

### Myogenesis and hypertrophy

CON/ECC^+^ induced significantly greater muscle hypertrophy compared with CON/ECC in the rehabilitation after ACL-R [5]. In accordance with this finding, the changes in the MSTN-mRNA levels after the 12-wk training period were significantly different between the training groups. MSTN is a negative regulator of muscle mass and reduces muscle growth by inhibiting myoblast proliferation and differentiation [18]. As an acute response to resistance exercise, a decrease in MSTN-mRNA expression has repeatedly been described in muscle biopsies obtained between 1 h and 24 h post-exercise [19,20]. The results of studies on the changes of steady-state MSTN-mRNA levels after long-term strength training are controversial. Decreases in MSTN-mRNA expression after several weeks of regular strength training were observed [21] as well as increases in MSTN-mRNA expression [22,23] or unchanged MSTN-mRNA levels [24,25]. Apparently, the time-point of the muscle biopsy sampling after the last bout of resistance exercise is of importance for the MSTN-mRNA expression. MSTN-mRNA expression was acutely downregulated between 1 hour and 24 hours (probably up to 48 hours) after resistance exercise [19], was shown to be still decreased after 3 days of detraining [26] and was significantly increased after 10 up to 90 days of detraining [26]. In the present investigation, muscle biopsies were obtained under resting conditions, 5–8 days after the last exercise bout with regular activity of daily living, but without exercises. Therefore, the mRNA expressions can be regarded as steady-state levels without obvious detraining effects.

The observation of a significant difference in the changes of the MSTN-mRNA levels between the training groups with a tendency towards a decrease after 12 weeks of CON/ECC^+^ is in contrast with the results of one of our previous studies on the effects of eccentric-overload strength training. In this previous study, we found a significant increase in MSTN-mRNA expression after 6 weeks of CON/ECC^+^ training while there was a tendency towards a decrease after conventional CON/ECC training [14]. This discrepancy, however, might be due to the quality of the investigated muscle, which now was a recovering atrophied muscle after ACL-R in contrast to a strength-trained muscle in the previous study. There is one other study on the effects of ACL injury on the MSTN-mRNA and MSTN-protein expression. In this other investigation, significantly elevated MSTN-mRNA and MSTN protein expression was observed and it was concluded that the induction of myostatin might be the key in the cause of muscle maladaptation with fibrotic tissue deposition, however, the subjects did not perform any strength training [11]. Therefore, we hypothesize that CON/ECC^+^ might not only have positive effects on the development of muscle hypertrophy but, in the long term, also on muscle quality. Further studies are needed to examine the effects of different strength training interventions on the atrophied quadriceps muscle of obviously reduced quality after ACL-R.

As MSTN acts by binding to the activin IIb receptor (ACVR2B) [27] the significant increase in ACVR2B-mRNA after CON/ECC^+^ training theoretically is disadvantageous for muscle growth processes and counteracting the decrease in MSTN-mRNA expression. However, the response of ACVR2B-mRNA to strength training has scarcely been investigated with inconsistent results [19–22] and the impact of the present finding remains unclear.

There were further indications for differences in the myogenic stimulus induced by CON/ECC^+^ or CON/ECC in the atrophied muscle after ACL-R. The mRNA expression of ARCV2B, MYF6 and ACTA1 were significantly increased after CON/ECC^+^ only, while a significant increase in IGF1-mRNA was only observed after CON/ECC. To our best knowledge, ACTA1-mRNA expression has not been investigated with regard to strength training effects in human skeletal muscle. ACTA1 is found in skeletal muscle and forms the sarcomere together with myosin and associated proteins. ACTA1 gene mutations have been shown to cause congenital myopathies [6]. The significant increase in ACTA1-mRNA expression after CON/ECC^+^ can be interpreted to be in accordance with muscle recovery after ACL-R. MYF6 (also known as myogenic regulator factor 4, MRF4) belongs to the myogenic regulatory family of transcription factors together with MYF5, MYOD and MYOG [28]. Acute responses in the mRNA expression of these factors have been described in some studies [24], however, controversial results were reported for steady-state mRNA expression with unchanged [24] or significantly increased [29] levels after 12 weeks of resistance training. In accordance with the results of one of our previous studies [14], MYF6-mRNA expression was significantly increased after CON/ECC^+^ only. IGF1-mRNA was the single marker-mRNA of muscle hypertrophy which only after CON/ECC showed a significantly increased expression. The results of several studies suggest that the IGF axis plays an important role in the mediation of anabolic effects in response to several weeks of strength training. Increases in the steady-state level of IGF1-mRNA have been reported after 5–8 weeks of CON/ECC [30,31] and after 6 weeks of CON/ECC^+^ [14]. However, after 12 weeks of conventional strength training, no increase in the steady-state levels of IGF1-mRNA was found [24].

### Contractility

Eccentric-overload strength training is regarded as a means to induce not only a stronger, but also a faster muscle phenotype due to the increased recruiting of the fastest type II fibers during the augmented eccentric load [12–14]. Surprisingly, CON/ECC^+^ strength training during rehabilitation after ACL-R lead to a significant increase in the proportion of the slow type I fibers. The significant upregulation of MYH2-mRNA, however, suggests that some more weeks of eccentric-overload training might induce a faster muscle phenotype with an increased type II fiber area. An increased eccentric load causes augmented mechanical stress which probably was reflected by the significantly increased expression of TTN-mRNA in the CON/ECC^+^ group only. Titin and titin-interacting proteins participate in the sensing of mechanical stress [32], but, for further interpretation, TTN splicing variants have to be investigated [33]. There was a trend for a significantly different regulation of the mRNA expression of the fast skeletal muscle troponin T (TNNT3) after CON/ECC^+^ and CON/ECC with a tendency for an increase after CON/ECC and nearly unchanged TNNT3-mRNA expression after CON/ECC^+^. The meaning of this finding is unclear as considerable variation in the splicing of the pre-mRNA encoding TNNT3 is crucial for skeletal muscle function [34,35]. However, splicing was not investigated in the present study. The impact of the slight differences between CON/ECC^+^ and CON/ECC in the changes of the mRNA expression of CAPN3, SGCA, and DYSF remains unclear. These marker-mRNAs, all belonging to the Dystrophin-Glycoprotein-Complex, have scarcely been investigated in human skeletal muscle with inconclusive results [36,37].

### Muscle wasting and atrophy

The differences in the expression of genes involved in skeletal myogenesis and skeletal muscle hypertrophy were not as clear-cut as expected. One reason might be ongoing processes of muscle atrophy and wasting as indicated by the significantly increased mRNA-levels of MAPK8 (JNK1), FBXO32 (atrogin-1, MAFbx) and TRIM63 (MuRF). MuRF1 (TRIM63) and atrogin-1 (FBXO32) belong to the exercise-responsive genes involved in the muscle cell ubiquitin/proteolysis pathway [38]. Increased levels of MAFbx- (FBXO32-) mRNA and a trend for an upregulation of MuRF- (TRIM63-) mRNA expression were reported in the atrophied human vastus lateralis muscle after 2 weeks of limb immobilization in healthy volunteers [20]. MuRF- (TRIM63-) mRNA expression was found to be increased in the sarcopenic vastus lateralis muscle of patients with chronic heart failure [39]. Both these mRNAs were downregulated after regular exercise. With regard to these results, a decrease in TRIM63- and FBXO32-mRNA expression could be expected in the regenerating vastus lateralis muscle after 12 weeks of supervised quadriceps strength training. In contrast, steady-state levels of FBXO32- and TRIM63-mRNA were significantly increased after 12 weeks of strength training without significant differences between CON/ECC^+^ and CON/ECC. These results might be explained by negative alterations within knee extension muscles which have been described after ACL injury [9–11]. It seems that also processes of atrophy and wasting were induced by strength training during remodeling of the quadriceps muscle after ACL-R. Also, the small increase in MAPK8- (JNK1-) mRNA expression, an inflammation marker related to kinase signaling pathways, points to this direction. To our best knowledge, there are no studies on MAPK8-mRNA expression in atrophied or trained human skeletal muscle. However, JNK1-protein abundance was found to be significantly increased in the vastus lateralis muscle of patients with knee osteoarthritis [40].

Furthermore, the significantly increased IKBKB- (IKKβ-) and HDAC5-mRNA levels, in the actually used array classified as marker-mRNAs for skeletal muscle contractility or skeletal myogenesis/skeletal muscle hypertrophy, respectively, should be looked at in the context of still persisting atrophy processes. After 2 weeks of limb immobilization, the increased expression of IKKβ-mRNA was assumed to lead to NFκB activation and reduced apoptosis [20]. In an animal study, the protein expression level of phosphorylated IKKβ was suggested as marker for skeletal muscle atrophy as a sustained significant decrease in phosphorylated IKKβ was observed from 2 to 28 days after denervation of rat gastrocnemius muscle [41]. Changes in the expression of HDAC5-mRNA in response to resistance training have, to our best knowledge, not been investigated yet. HDAC5 belongs to the class IIa histon deacetylases. In human skeletal muscle, HDAC5 is exported from the nucleus during exercise thereby removing its transcriptional repressive function for myocyte enhancer factor-2 proteins [42]. HDAC5-mRNA expression was shown to be increased after 10 days of immobilization in mice tibialis anterior muscle [43]. The significantly increased HDAC5-mRNA expression after CON/ECC might therefore, hypothetically, point to still ongoing atrophying processes in the investigated muscle counteracting muscle recovery.

Additionally, the expression changes of ADIPOQ-mRNA are interesting with regard to dysfunctional muscle after ACL-reconstruction. ADIPOQ-mRNA expression was found to be significantly increased in boys with Duchenne muscle atrophy [44] and in the concave-sided muscle in patients with severe adolescent idiopathic scoliosis compared to the convex-sided muscle with the higher amplitude of motor unit potentials in electromyography activity measurements [45]. The strong tendency towards a different regulation of the ADIPOQ-mRNA expression in both training groups might be interpreted as a positive effect of the CON/ECC^+^ strength training on fatty infiltration of the vastus lateralis muscle. However, as ADIPOQ-mRNA was only expressed in half of the subjects of the CON/ECC training group and in 2/3 of the subjects of the CON/ECC^+^ training group, these results must be interpreted with caution.

### Effects of different types of autograft

Some effects of the type of autograft on muscle regeneration during quadriceps strength training emerged in the expression changes of 6 marker mRNAs involved in skeletal myogenesis, skeletal muscle hypertrophy and in skeletal muscle contractility, respectively. The expression of these marker mRNAs showed a decrease or smaller increase in the QUAD subjects compared with the SEMI subjects after the 12-wk quadriceps strength training. These results support our previous observation that recovery of the quadriceps muscle after ACL-R is affected by the type of autograft [5]. Accordingly, significant effects on muscle strength by autograft type were reported in another study one year after ACL-R with more pronounced deficits in quadriceps muscle strength after QUAD compared with SEMI [3].

### Limitations

It is a draw-back of the present study and might have caused some variation in the mRNA levels that the biopsies could not be obtained on exactly the same time-point after the last exercise bout in all subjects due to the subjects´ individual, not study-related time schedules. The subjects´ activities of regular daily living probably also differed to some extent. All our subjects were either back to work after the ACL-surgery or students. It was difficult to make them follow a stringent time table of training and testing for more than 12 weeks. Standardization of training and testing is a challenge in real-life studies and certainly is a reason for the few studies in the field and for low subject numbers. Compared to the–to our best knowledge–only 2 other investigations on the effects of ACL injury on morphology and gene expression in quadriceps muscle, the subject number of our study (n = 31) was quite high. In these other studies, the effects of ACL injury on quadriceps muscle quality were investigated in 9 and 10 subjects, respectively, and these subjects were not subjected to strength training for several weeks [4,9–11]. However, low subject number always is a cause for the lack of clear-cut significant differences in gene expression changes in human skeletal muscle. Another draw-back is the lack of a control group without training. When the study was designed, we considered the positive effects of strength training during rehabilitation after ACL-R and planned the CON/ECC group as the control group for the CON/ECC^+^ group. Unfortunately, we did not anticipate the large inter-individual variation introduced by the factors already mentioned.

## Conclusion

The significant difference in the change of the MSTN-mRNA expression between the CON/ECC and the CON/ECC^+^ group and the significant increases in MYF6- and ACTA1-mRNA levels only after CON/ECC^+^ probably reflect the previously reported stronger hypertrophic stimulus of CON/ECC^+^ compared with CON/ECC, also during rehabilitation after ACL-R. The expected shift towards a faster muscle phenotype might be indicated by the significant increase in the MYH2-mRNA expression, the enhanced mechanical stress by the significant increase in TTN-mRNA expression after CON/ECC^+^ only. However, we did not find the expected clear differences between CON/ECC^+^ and CON/ECC in gene expression changes. The lack of clear-cut differences might be due to a great inter-individual variation, at least partly induced by ongoing processes of muscle wasting and atrophy as well as by the different types of autografts.

## Supporting information

S1 Fig Effects of 12 wk one-legged CON/ECC or CON/ECC^+^ leg-press training on muscle cross sectional area (MCSA), fiber cross sectional area (FCSA) and fiber type distribution. Mean ± SE are shown. #: Significant group x time effect (p < 0.05); §: Significant time effect (p = 0.001); **: p < 0.01, ***: p < 0.001 compared with the respective value before training.
(TIF)

S1 File Data set. Individual data points of all subjects.
(XLSX)
